# Detection of PRRSV-1 in tongue fluids under experimental and field conditions and comparison of different sampling material for PRRSV sow herd monitoring

**DOI:** 10.1186/s40813-024-00370-0

**Published:** 2024-05-19

**Authors:** Sophie Dürlinger, Heinrich Kreutzmann, Christine Unterweger, Vera Martin, Flora Hamar, Christian Knecht, Angelika Auer, Katharina Dimmel, Till Rümenapf, Alfred Griessler, Thomas Voglmayr, Roland Maurer, Alexander Oppeneder, Andrea Ladinig

**Affiliations:** 1 Clinical Department for Farm Animals and Food System Science, Clinical Centre for Population Medicine in Fish, Pig and Poultry, University of Veterinary Medicine Vienna, Veterinaerplatz 1, 1210 Vienna, Austria; 2 GD Animal Health Service, P.O. Box 9, 7400 AA Deventer, The Netherlands; 3 Department of Biological Sciences and Pathobiology, Institute of Virology, University of Veterinary Medicine Vienna, Veterinaerplatz 1, 1210 Vienna, Austria; 4 Traunkreis Vet Clinic GmbH, Grossendorf 3, 4551 Ried im Traunkreis, Austria

**Keywords:** Porcine reproductive and respiratory syndrome virus, PRRSV, AUT15-33, Vertical transmission, Reproductive model, Processing fluids, Tongue tissue, Tongue fluids

## Abstract

**Background:**

Infection with porcine reproductive and respiratory syndrome virus (PRRSV) leads to significant economic losses worldwide. One of the initial measures following an outbreak is to stabilise the herd and to prevent vertical transmission of PRRSV. The objective of this study was to detect PRRSV in different sampling material, both in an experimental model and on a commercial piglet producing farm, with a focus on evaluating the suitability of tongue fluid samples.

**Results:**

In the experimental model, PRRSV negative pregnant gilts were infected with PRRSV-1 AUT15-33 on gestation day 85 and necropsy of gilts and foetuses was performed three weeks later. 38.3% of individual foetal serum and 39.4% of individual foetal thymus samples were considered PRRSV RT-qPCR positive. Tongue fluids from individual foetuses showed a 33.0% positivity rate. PRRSV RNA was detected in all but one sample of litter-wise pooled processing fluids and tongue fluids. In the field study, the investigated farm remained PRRSV positive and unstable for five consecutive farrowing groups after the start of the sampling process. Tongue fluid samples pooled by litter in the first investigated farrowing group had a 54.5% positivity rate, with the overall highest viral load obtained in the field study. In this farrowing group, 33.3% of investigated litter-wise pooled processing fluid samples and all investigated serum samples (pools of 4–6 individuals, two piglets per litter) were considered positive. Across all investigated farrowing groups, tongue fluid samples consistently showed the highest viral load. Moreover, tongue fluid samples contained the virus in moderate amounts for the longest time compared to the other investigated sampling material.

**Conclusion:**

It can be concluded that the viral load in individual foetuses is higher in serum or thymus compared to tongue fluid samples. However, litter-wise pooled tongue fluid samples are well-suited for detecting vertical transmission within the herd, even when the suspected prevalence of vertical transmission events is low.

## Background

Porcine reproductive and respiratory syndrome virus (PRRSV) still represents an economically significant problem in pig production worldwide, since it is responsible for economic losses in both breeding- and growing pig herds [1–3]. To categorise a breeding herd based on its PRRSV status and thus evaluate the need or the success of a PRRSV control program, regular PRRSV monitoring is needed. A herd classification system, which was described by the American Association of Swine Veterinarians (AASV) in 2010, is based on serum samples collected from weaning-age pigs, tested by reverse transcription quantitative PCR (RT-qPCR) [4]. Recently developed population-based methods for PRRSV monitoring, such as investigation of processing fluids, were one of the reasons for the modification of the classification system in 2021 [5].

Since collection of blood samples is time consuming, requires at least one trained person and a veterinarian and causes additional stress for the piglets, more practical, less time- and cost-consuming sampling methods are investigated. Collection of oral fluids for subsequent PRRSV analysis via PCR or ELISA is a non-invasive, time- and cost- saving monitoring method, which is often used in growing and adult pigs to detect PRRSV nucleic acids or PRRSV specific antibodies [6]. It has been shown that less PRRSV RNA was found in litter-/pen- based oral fluids than in serum of individual piglets [7, 8]. De Regge et al. demonstrated, that the probability to detect PRRSV in pen-based oral fluids correlated with the percentage of serum PCR-positive pigs [9]. However, to assess stability within a sow herd, it is necessary to sample newborn piglets to detect vertical PRRSV transmission from dams to their foetuses. Collection of oral fluid samples from suckling piglets is not as simple as it is from older pigs [10]. In addition, oral fluids were shown to be less suitable than serum to detect PRRSV early after infection [11].

In 2018, PRRSV monitoring was improved by the use of processing fluids, serosanguinous fluids recovered at castration and tail docking [12]. As an aggregate sample, which can be easily collected by farm staff, processing fluids provide testing a higher number of suckling piglets. The results of Lopez et al. showed that the probability of PRRSV RNA detection by PCR in processing fluids was higher than in the 30 matching serum samples, which were tested in pools of five [12]. Lopez et al. described that pooling of processing fluids from several litters increased the probability of PRRSV detection at herd level compared to testing the same number of individual litter samples due to the higher number of potential PRRSV positive piglets in the sample [13]. The detection of PRRSV in aggregated samples depends on the viral load of the positive piglets and thus more litters can be pooled during the initial stage of infection than a few months after an acute PRRSV outbreak [14]. Processing fluids were demonstrated to be a reliable tool to monitor PRRSV in herds undergoing virus elimination after implementing herd closure and mass vaccination with a PRRS modified live virus vaccine [15]. Overall, processing fluids seem to be a practical, time- and cost-efficient aggregated sample material to monitor PRRSV in newborn piglets.

Nonetheless, the prohibition of pig tail docking in the European Union, which only allows this practice under certain conditions and according to an indication, but not routinely, is one reason why new, innovative but also time- and cost-efficient PRRSV monitoring methods are still under investigation [16, 17]. Apart from that, surgical castration without anaesthesia is already prohibited in many European countries, and alternatives to the surgical castration of male pigs are the subject of social discussion [18]. Therefore, alternative sampling methods are needed. Baliellas et al. described the use of tongue tips from stillborns and piglets which die during the lactating period as an aggregated sample for PRRSV monitoring and claimed that the investigation of tongue exudate is more sensitive than the investigation of the corresponding serum samples [19]. Similar results were obtained in a study of Machado et al. who investigated the presence of PRRSV RNA in serum samples, processing fluids, family oral fluids, and tongue tip samples by RT-PCR [20].

The aim of the present study was to evaluate the suitability of foetal tongue fluids compared to other sampling material for the detection of PRRSV-1 by RT-qPCR, both under experimental conditions and in the field.

## Methods

### Study design

Two different experiments were performed: (1) A study under experimental conditions to compare the PRRSV-1 viral loads in individual foetal tongue fluids with the viral loads in individual foetal serum and thymus tissue samples. Additionally, litter-based samples were evaluated. For this purpose, viral loads in litter-wise pooled tongue fluids were compared to viral loads in processing fluids pooled by litter. (2) A field study to assess whether tongue fluids of stillborn piglets and piglets that died in the first days of life represent a suitable sample material to monitor PRRSV-1 positive breeding farms after an acute PRRSV-1 outbreak.

### Study under experimental conditions

26 pregnant gilts (Danish genetics) from a farm in Lower Austria with negative status for PRRSV were included in this experiment. The negative status of these animals was verified by pre-monitoring via ELISA and RT-qPCR. The gilts were vaccinated against porcine parvovirus in combination with erysipelas (Parvoruvac®, Ceva Santé Animale, France) and against influenza virus (Respiporc FLU3®, Ceva Santé Animale) and porcine circovirus type 2 (Ingelvac CircoFLEX®, Boehringer Ingelheim Vetmedica GmbH, Germany). One week prior to challenge, the gilts were transferred to the University of Veterinary Medicine Vienna, where they were housed in the biosafety level 2 isolation unit.

Experimental infection was performed on day 85 of gestation. PRRSV-1 subtype 1 strain AUT15-33 (gene bank accession number MT000052.1) was intranasally inoculated with a total dose of 5 × 10^5^ TCID_50_. The gilts were retained by use of a snare, and the virus was vaporised directly into the nostrils (5 mL of cell culture supernatant including the virus) using a mucosal atomisation device. At termination, gilts and their litters were euthanised and necropsies of gilts and foetuses were performed between gestation days 104–110. Processing fluids including testes from all male foetuses and tail tips from male and female foetuses were pooled per litter in sterile sampling bags (Whirl–Pak®, Nasco Sampling, Wisconsin, USA) and examined for PRRSV-specific genome fragments by RT-qPCR. Furthermore, the tongues of all foetuses were removed using sterile scissors and forceps, and simultaneously litter-wise pooled.

In five litters, further investigations of the foetuses were conducted. The preservation status from a total of 104 foetuses was evaluated and classified as viable (VIA), meconium-stained (MEC), decomposed (DEC), autolysed (AUT) or mummified according to Ladinig et al. [21]. In addition, MEC foetuses were further divided into MEC1 and MEC2 based on meconium distribution according to Malgarin et al. [22]. To assess the viral load in individual sample material, foetal thymus and foetal serum were examined by RT-qPCR. Furthermore, the tongue of these foetuses was divided into two parts: A small piece was used for litter-wise pooling, whereas the major part of the tongue was stored individually in pathology tubes with enclosed screw caps (KABE-Labortechnik GmbH, Nümbrecht-Elsenroth, Germany) for further analysis.

All samples were frozen to − 20 °C. For further processing of the tongue tissue, samples were thawed, and the liquid obtained was used for RT-qPCR. In most of the foetuses, the fluid obtained by freezing and thawing the tongues was sufficient to perform RT-qPCR (n = 88).

### Field study

In the field study, a piglet producing farm was monitored over ten farrowing groups following an acute PRRS outbreak. The farm is a conventional farm with approximately 120 breeding sows and is managed in a three-week batch farrowing rhythm with a weaning age of 28 days. Gilts are purchased from a farm with negative status for PRRSV. In March 2022, PRRSV-1 was detected for the first time in this previously PRRSV negative farm. At this time point, the sows showed symptoms such as fever, coughing, and an increased return to oestrus rate. Piglets were born weak and showed dyspnoea and diarrhoea. In one farrowing batch, only 50 piglets out of 21 litters survived until weaning. Immediately after the first PRRSV-1 detection, the farm started vaccinating the sows with ReproCyc® PRRS EU (Boehringer Ingelheim Vetmedica GmbH). The second vaccination of the entire sow herd was performed four weeks later. Re-vaccinations of the sows were implemented with 2.5–3 months intervals. Vaccination of the piglets with PRRSFlex® EU (Boehringer Ingelheim Vetmedica GmbH) was started two months after initial virus detection according to the manufacturer’s instructions.

For monitoring purposes, litter-wise pooled tongue tissue samples from stillborn piglets and piglets that died in the first days of life were collected after implementing the sow herd vaccination. Additionally, processing fluids were gathered from all piglets and pooled per litter. Furthermore, serum samples from two piglets per litter at three weeks of life were examined in pools of four to six piglets, and oral fluid samples from piglets after weaning (5th week of life) were examined for PRRSV by RT-qPCR. All samples were frozen to − 20 °C.

### Virological analysis—PRRSV RT-qPCR and sequencing

Liquid samples and tissue lysates were thawed at room temperature, then vortexed for 10 s and centrifuged at 16,000 × g for one minute. 140 µL of supernatant was extracted employing the QIAamp® Viral RNA Mini Kit in a QIAcube® (QIAGEN, Germany) and RT-qPCRs were performed using Luna® Universal One-Step RT-qPCR Kit (New England BioLabs®, Inc., USA) on a qTower^3^ G Realtime machine (Analytic Jena GmbH, Germany) following the manufacturer’s instructions. Primers (sense: 5′-TTTATTCTCGACTCCATCCAACC-3′, antisense: 5′-AAAGTTGGCGCTGCTCA-3′) and probe (FAM-5′-TCTTCTTGTGASCACGATTCGCCG-3′-BHQ1) were designed to amplify a 98 bp fragment of the PRRSV-1´s conserved ORF1a region. Samples were considered positive if the RT-qPCR demonstrated more than 10^4^ genome equivalents (GE)/mL or g per sample. Blanks consisting of sample-free extracts as well as no template controls served as negative controls. Beta-actin mRNA RT-qPCR was performed for each sample extract to exclude PCR inhibiting substances [23].

### Statistical analysis

Statistical analysis was conducted using RStudio [24]. Measurement data are expressed as mean ± standard deviation (SD). A Spearman correlation coefficient was calculated to assess the relationship between viral loads in different sample types, including tongue fluids and serum at the individual animal level, tongue fluids and thymus at the individual animal level, and pools of processing fluids and tongue fluids.

## Results

### Experimental trial—Viral loads in individual serum, thymus and tongue fluid samples

**Fig. 1:**
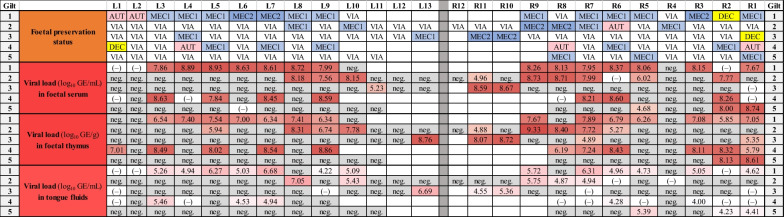
Investigation of individual foetuses in the experimental setup. Foetal preservation status (VIA = viable; MEC = meconium-stained; DEC = decomposed; AUT = autolysed) and viral load in serum, thymus and tongue fluids of individual foetuses of five infected gilts. Foetuses were numbered according to their location in the uterus with the closest one to the ovary named “L1” in the left horn and “R1” in the right horn. Each line represents one litter. For the foetal preservation, colours represent the different categories (VIA in white, MEC in light blue [MEC1] or dark blue [MEC2], DEC in yellow and AUT in red). Viral load is displayed in genome equivalents per mL serum or tongue fluids or g thymus tissue. The red filling represents the viral load (light red to dark red–low to high amount). Grey filling with [neg.] indicates that the investigated sample was below the detection limit of the PRRSV-1 ORF1 RT-qPCR and can be considered as negative. (–) = not sampled

**Fig. 2:**
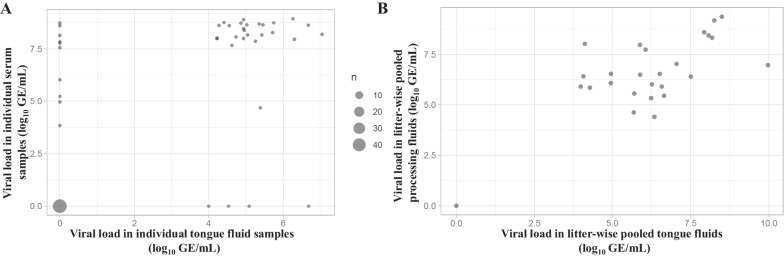
Correlation of PRRSV viral loads between sample types. (**A**) Correlation of viral loads between individual tongue fluids and serum samples and (**B**) pooled tongue fluids and processing fluid samples from the experimental setup

From the foetuses of the five infected gilts, foetal preservation status was assessed and serum, thymus and fluids from individual tongues were collected from each foetus (n = 104) for RT-qPCR investigation. The results are displayed in Fig. 1 and Table 1. The foetal preservation status of individual foetuses ranged from VIA to AUT with the highest percentage of foetuses categorised as VIA (62%), followed by foetuses categorised as MEC1 (23%), MEC2 (7%), AUT (6%) and DEC (3%). The viral load in foetal serum ranged from below the cut-off to 8.93 log_10_ GE/mL serum, with 36 positive (38.3%) samples. Ten samples could not be collected due to the impaired foetal preservation status (DEC and AUT) and the lack of serum. The mean viral load of positive samples was 7.97 ± 1.06 (SD) log_10_ GE/mL serum. For foetal thymus, the viral load ranged from below the cut-off to 9.33 log_10_ GE/g thymus tissue, with 41 positive (39.4%) and 63 negative samples. The mean viral load of positive samples was 7.29 ± 1.17 log_10_ GE/g tissue. In tongue fluids of individual foetuses, the viral load ranged from below the cut off to 7.05 log_10_ GE/mL tongue fluids, with 29 positive (33.0%) and 59 negative samples. Sixteen samples could not be collected due to the impaired foetal preservation status (DEC and AUT) and the lack of fluids of the individual tongue samples. The mean viral load of positive samples was 5.20 ± 0.79 log_10_ GE/mL tongue fluids. The comparison of PRRSV viral loads in tongue fluids and serum at individual animal level revealed a positive correlation with a Spearman correlation coefficient of 0.71 (Fig. 2A). For individual tongue fluid and thymus samples, the Spearman correlation coefficient was 0.76. The percentage agreement between the results (RT-qPCR positive/negative) obtained from the three different sample matrices is displayed in Table 2.

**Table 1:** Results of PRRSV RT-qPCR investigations from the experimental setup

Sample material	Investigated samples (n)	RT-qPCR positive (n)	Maximum viral load (log_10_ GE/mL or g)	Mean viral load and standard deviation of positive samples (log_10_ GE/mL or g)
Serum from individual foetuses	94	36 (38.3%)	8.93	7.97 ± 1.06
Thymus from individual foetuses	104	41 (39.4%)	9.33	7.29 ± 1.17
Tongue fluids from individual foetuses	88	29 (33.0%)	7.05	5.20 ± 0.79
Processing fluids pooled by litter	26	25 (96.1%)	9.36	6.77 ± 1.36
Tongue fluids pooled by litter	26	25 (96.1%)	9.97	6.49 ± 1.97

**Table 2:** Percentage agreement in RT-qPCR results (positive/negative) in different sample matrices from individual foetuses

	Serum (%)	Thymus (%)	Tongue fluids (%)
Serum	100.0	90.4	85.1
Thymus	90.4	100.0	89.8
Tongue fluids	85.1	89.8	100.0

### Pooled tongue fluid and processing fluid samples per litter

In total 25 processing fluid samples and the corresponding 25 tongue fluid samples were considered RT-qPCR positive. Results are displayed in Fig. 2B and Table 1. In positive samples, viral load was 6.77 ± 1.36 (SD) and 6.49 ± 1.97 log_10_ GE/mL in processing fluid and tongue fluid samples, respectively. The comparison of the two sampling types revealed a positive correlation with a Spearman correlation coefficient of 0.52 (Fig. 2B).

### Field study

**Fig. 3:**
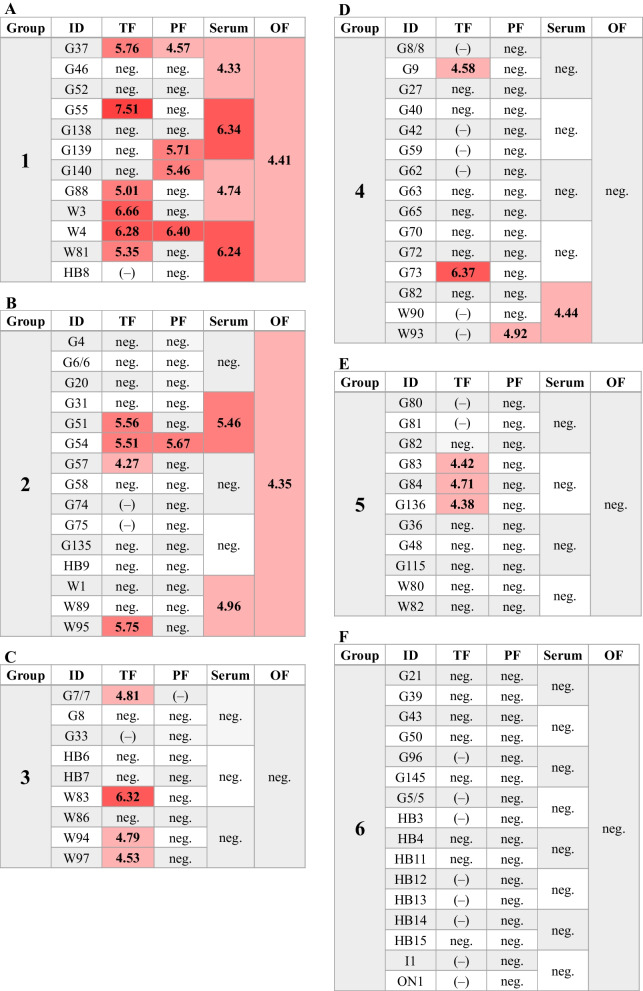
Visualisation of the results from the field trial. Results from farrowing group 1 (**A**) to farrowing group 6 (**F**). ID = sow identification number; TF = tongue fluids; PF = processing fluids; OF = oral fluids; (–) = not investigated; neg. = below detection limit of the RT-qPCR. Viral load is illustrated as log_10_ transformed genome equivalents per g thymus tissue or mL serum/fluid. The red filling represents the viral load (light red to dark red–low to high amount)

In total, the investigated farm was PRRSV positive unstable for five consecutive farrowing groups after the beginning of the sampling process. Results are displayed in Fig. 3. In farrowing group 1 (Fig. 3A), tongue fluid samples were collected in 11/12 litters, with a 55% positivity rate. In these litter-wise pooled tongue fluid samples, the overall highest viral load of the field study was obtained (7.51 log_10_ GE/mL). In this farrowing group, 4/12 investigated processing fluid samples and all investigated serum samples were considered positive. One oral fluid sample was collected from the whole group of weaned piglets, with a positive result close to the cut-off value of the PCR protocol.

In the consecutive farrowing group three weeks later (Fig. 3B), tongue fluid samples were collected from 13 out of 15 litters, 27% of them were positive by RT-qPCR. Only one of the processing fluid samples was considered positive, whereas 2/5 serum pools showed a positive RT-qPCR result. The oral fluid sample was also positive, with a low viral load of 4.35 log_10_ GE/mL. In the third farrowing group (Fig. 3C), consisting of nine litters, 4/8 tongue fluid samples were positive. All other samples were considered negative. Fifteen litters were present in farrowing group 4 (Fig. 3D). Two out of nine investigated tongue fluid samples were positive, as well as 1/15 processing fluid samples and 1/5 serum samples. The oral fluid sample was negative. In farrowing group 5 (Fig. 3E), only tongue fluid samples were positive, with a positivity rate of 33%. All samples of the consecutive farrowing groups were considered negative by RT-qPCR (Fig. 3F).

## Discussion

Within the last years, newly developed population-based methods for PRRSV monitoring offered new diagnostic opportunities with less effort and costs [5, 25]). The eligibility and the effect of pooling of processing fluid samples is well established in the field [13–15, 25]. Initial studies suggested similar practicability for tongue fluid samples [19, 20]. In all studies, tongue tissue samples were frozen (− 20 °C) until examination. However, different methods for sample processing were used. In the study of Machado et al., the exudate from tongue tips was extracted by adding phosphate buffered saline solution (PBS) to the tongue tips, followed by a homogenisation step [20]. In the study of Baliellas et al., bags with thawed tongue tissues were homogenised, then the exudate at the bottom of the bag was collected [19]. In the current study, samples were thawed, and the liquid was directly obtained from the tubes or the sterile sampling bags. The comparison of different processing methods was not within the scope of the study; however, the results suggest that the homogenisation step and the washing with PBS is not necessarily needed to obtain enough liquid to be used in RT-qPCR for PRRSV detection.

For the experimental setup, the virulent strain AUT15-33 was used. This strain was first isolated in 2015 in a piglet-producing farm with 80 sows in Lower Austria. After infection, losses in foetuses and suckling piglets raised up to 90%, and the return to oestrus rate peaked at 60% [26]. The virulence of this strain was demonstrated experimentally, both in the respiratory and reproductive model [27–30]. In the reproductive model, a comparable experimental setup to the current study was used. Challenge with AUT15-33 was performed at gestation day 84. Twenty-one days later, the number of infected foetuses was highly variable between litters. The foetal preservation status was impaired in 44% of the foetuses compared to 38% in the current study; however, tongue fluids were not investigated [28]. In the current study, it was demonstrated that there is a strong positive correlation in the PRRSV RNA load of individual tongue fluid samples compared to reference samples, i.e. serum and thymus. Furthermore, the comparison of results (RT-qPCR positive/negative, Table 2) from individual matrices indicates a high level of agreement.

Nevertheless, the aim of sampling should be defined. If the aim is PRRSV monitoring of vertical transmission within the herd, tongue fluid samples seem to be a suitable sampling material. Although the experimental setup has shown that the viral load is higher in serum and thymus samples than in the corresponding tongue fluid samples and therefore more suitable for sequencing, it should be kept in mind that stillborn piglets or weak born piglets that die within the first days of life are the ones from which tongue tissue is collected. These are the piglets with higher probability of being PRRSV positive, which could be demonstrated in the current setup of the field trial. In all investigated farrowing groups, tongue fluid samples showed the highest viral load of all investigated samples in the respective group. In addition, tongue fluid samples contained virus in moderate amounts for the longest time compared to processing fluids, oral fluids and serum samples. It should be emphasized that this is the first study to compare viral loads in individual piglet tongue fluid samples to serum and thymus samples in an experimental setting. Additionally, only one conventional farm was used to evaluate the reliability of tongue fluid samples following a natural PRRSV outbreak. The study design is exploratory in nature. Therefore, a more in-depth statistical analysis of the available dataset was deliberately omitted to mitigate the risk of the “HARKing” effect (hypothesizing after the results are known) [31]. The collected data provide a basis for subsequent studies to further confirm the reliability of tongue fluids as a method for monitoring PRRSV in sow herds.

Population-based methods for PRRSV monitoring (processing fluids and family oral fluids) are part of the updated PRRSV herd classification system of the AASV, either as a substitute for serum samples or as alternate sampling material [5]. The suspected low prevalence of infected animals in herds that want to promote into the “positive stable” category led to a modification of the sample size. Sampling a minimum of 60 pigs per investigation, analysed in pools of 10 pigs, should result in a better PRRSV detection of a lower prevalence. With the alternate use of processing fluids the sample size can be reduced [5]. Up to now, tongue fluid samples are not mentioned in the AASV classification system. The results of the field trial suggest that tongue fluid samples can detect vertical transmission of virus, even when the suspected prevalence of vertical transmission events is low. Further studies in the field are needed to support the findings of this study.

## Conclusion

Based on the results of the experimental study, it can be concluded that in individual foetuses the viral load is higher in serum or thymus samples compared to the respective tongue fluid sample. Nevertheless, when examining litter-wise tongue fluid samples in the field study, they proved effective in identifying vertical transmission within the herd, even under conditions of low suspected prevalence of vertical transmission events. Based on these findings, it can be concluded that tongue fluids serve as a suitable sample matrix for monitoring purposes. The results can be highly relevant for practicing veterinarians in the field, especially when it comes to implementing PRRS monitoring in sow herds.

## Data Availability

The datasets used and analysed during the current study are available from the corresponding author on reasonable request.

## Abbreviations

AASV: American Association of Swine Veterinarians
AUT: Autolysed
DEC: Decomposed
GE: Genome equivalents
MEC: Meconium-stained
PBS: Phosphate buffered saline solution
PRRSV: Porcine reproductive and respiratory syndrome virus
RT-qPCR: Reverse transcription quantitative PCR
SD: Standard deviation
VIA: Viable
